# Stimulation of peroneal nerves reveals maintained somatosensory representation in transtibial amputees

**DOI:** 10.3389/fnhum.2023.1240937

**Published:** 2023-09-07

**Authors:** Caroline Ritter, Maria Geisler, Kathrin R. Blume, Sandra Nehrdich, Gunther O. Hofmann, Hanna Koehler, Wolfgang H. R. Miltner, Thomas Weiss

**Affiliations:** ^1^Department of Clinical Psychology, Institute of Psychology, Friedrich Schiller University Jena, Jena, Germany; ^2^Clinic for Psychosomatics and Psychotherapy, Jena University Hospital, Jena, Germany; ^3^Institute of Psychosocial Medicine, Psychotherapy and Psychooncology, Jena University Hospital, Jena, Germany; ^4^Berufsgenossenschaftliche Kliniken Bergmannstrost Halle/Saale, Halle, Germany; ^5^Department of Trauma, Hand and Reconstructive Surgery, University Hospital Jena, Jena, Germany; ^6^Biomagnetic Center, Department of Neurology, University Hospital Jena, Jena, Germany

**Keywords:** phantom limb pain (PLP), somatosensory cortex (SI), peroneal nerve stimulation, somatosensory evoked fields (SEF), MEG, transtibial amputation

## Abstract

**Introduction:**

Several studies have found changes in the organization of the primary somatosensory cortex (SI) after amputation. This SI reorganization was mainly investigated by stimulating neighboring areas to amputation. Unexpectedly, the somatosensory representation of the deafferented limb has rarely been directly tested.

**Methods:**

We stimulated the truncated peroneal nerve in 24 unilateral transtibial amputees and 15 healthy controls. The stimulation intensity was adjusted to make the elicited percept comparable between both stimulation sides. Neural sources of the somatosensory-evoked magnetic fields (SEFs) to peroneal stimulation were localized in the contralateral foot/leg areas of SI in 19 patients and 14 healthy controls.

**Results:**

We demonstrated the activation of functionally preserved cortical representations of amputated lower limbs. None of the patients reported evoked phantom limb pain (PLP) during stimulation. Stimulation that evoked perceptions in the foot required stronger intensities on the amputated side than on the intact side. In addition to this, stronger stimulation intensities were required for amputees than for healthy controls. Exploratorily, PLP intensity was neither associated with stimulation intensity nor dipole strength nor with differences in Euclidean distances (between SEF sources of the healthy peroneus and mirrored SEF sources of the truncated peroneus).

**Discussion:**

Our results provide hope that the truncated nerve may be used to establish both motor control and somatosensory feedback via the nerve trunk when a permanently functional connection between the nerve trunk and the prosthesis becomes available.

## 1. Introduction

Amputation in adults is associated with the remapping of primary somatosensory cortical representations (Merzenich et al., 1983; Flor et al., 1995). Another relatively frequent sequela of amputation is phantom limb pain (PLP), which affects ~70% of amputees of a limb (Ehde et al., 2000; Gallagher et al., 2001; Ephraim et al., 2005). As PLP is often unpredictable and strong, it impairs almost all everyday activities and therefore represents a significant burden for most amputees, in addition to the functional disability (Dillingham et al., 2001; Murray and Fox, 2002). Understanding the mechanisms that drive alterations of the somatosensory nervous system after amputation could inform rehabilitation approaches. Some studies associated PLP with SI remapping after amputation and suggested the development of maladaptive plasticity (Flor et al., 1995, 2006; Kuner and Flor, 2017). This SI reorganization was mainly investigated with magnetoencephalography (MEG). They analyzed somatosensory magnetic fields (SEFs) in response to peripheral bottom-up stimulation of body parts that neighbor the cortical representation of the amputated limb, e.g., by stimulating the lip in case of arm amputation (Flor et al., 1995) or a neighboring finger in case of finger amputation (Weiss et al., 1998, 2000). The theoretical explanation of maladaptive plasticity was then recently challenged by studies that employed functional magnetic resonance imaging (fMRI) to observe somatosensory processing (Makin et al., 2013a,b). Makin et al. (2013b) concluded that loss of sensory input is associated with reduced functional and structural representations of the deafferented cortex; however, PLP is associated with increased representations of the phantom limb in SI that are potentially stabilized by nociceptive input. This study used an analysis technique to optimize separated representations.

Unexpectedly, the somatosensory representation of the deafferented limb has rarely been tested directly by measuring cortical somatosensory responses to bottom-up stimulation of the deafferented nerve, which had formerly supplied the primary somatosensory representation of the lost limb. Bottom-up stimulation could be achieved by electrical stimulation of truncated nerves that formerly supplied the lost body part using well-established procedures for the localization of central representations of intact body parts such as the hand or the leg (Kaukoranta et al., 1986; Huttunen et al., 2006; Cruccu et al., 2008; Baumgartner et al., 2010). The results of such studies would both potentially add knowledge about mechanisms driving PLP and inform interventions that use somatosensory feedback from the prosthesis (Dietrich et al., 2012, 2018b) or use longer-lasting direct stimulation of the truncated nerves for rehabilitation purposes (Raspopovic et al., 2014; Raspopovic, 2020).

The research objective of the study was to test whether a preserved representation in a deprived area of the cortex in the SI of lower limb amputees is a functional correlate of peripheral nerve stimulation and PLP intensity. We tested whether (1) it is possible to elicit a somatosensory percept of the missing limb by electrical transcutaneous stimulation of the peroneal nerve. If this is possible, we tested whether (2) it is possible to fit dipoles in the deprived cortex. Furthermore, we explored whether (3) the intensity needed to stimulate the truncated peroneal nerve differs from the intact peroneal nerve in amputees or from matched healthy volunteers (controls), and (4) whether the dipole characteristics of the affected side (AS) of the patients differ from the intact side and controls. Finally, we explored whether (5) dipole strength and stimulation intensity depend on PLP intensity.

## 2. Materials and methods

### 2.1. Experimental design

We carried out a laboratory experiment as a two-factor mixed design comprising the within-subject factor Stimulation Side (affected side, AS; non-affected side, NAS) and the between-subject factor Group (patients; controls). Questionnaires were used to assess phantom limb pain. Somatosensory-evoked magnetic fields were measured with magnetoencephalography (MEG) during transcutaneous stimulation. Blinded assessment of outcomes was realized by blinding the researcher, who analyzed ECDs, to the pain level of participants.

### 2.2. Participants

Criteria for the inclusion of patients were transtibial amputation and the use of lower limb prosthesis. Twenty-four unilateral lower limb amputees (eight females, mean age = 54.0 years ± 11.8, range: 25–76) were included into the study. Additionally, we recruited 15 controls (four females, mean age = 48.9 years ± 12.0, range: 24–64) not differing in age and sex distribution from patients. All subjects gave written informed consent to participate in the study. The characteristics of amputees are shown in Table 1. All subjects provided written informed consent to participate in this study. The study has been approved by the Ethics Committee of the University Hospital Jena (No. 1312-05/04).

**Table 1:** Clinical and demographic characteristics of subjects with lower limb amputation.

	**Patient characteristics**				**Pain characteristics**			
	**Age in years**	**Time since amputation in months**	**Side of amputation**	**Cause of amputation**	**VAS**_24h_	**magnitude**	**frequency**	**MPI**
1	40–50	152	R	Trauma	2.1	0.53	4	
2	40–50	24	L	Trauma	8.2	8.2	1	6.00
3	60–70	263	R	Trauma	0	0	5	
4	50–60	188	R	Sepsis following trauma	2.0	2.0	1	1.00
5	40–50	291	L	Trauma	4.9	4.9	1	1.00
6^*^	40–50	72	L	Trauma	6	6	1	1.67
7	50–60	2	R	Sepsis	5.7	5.7	1	1.67
8	60–70	45	L	Trauma	8.0	8.0	1	5.00
9	20–30	14	R	Trauma	0	0	3	0.33
10	60–70	41	L	Sepsis	8.9	8.9	1	3.00
11	40–50	6	L	Trauma	0	0	3	3.33
12^*^	50–60	186	R	Trauma	0	0	n.a.	0.00
13	50–60	408	L	Trauma	4.7	4.7	1	3.00
14	60–70	39	R	Trauma	1.7	0.6	3	2.33
15	20–30	21	L	Trauma	6.1	2.0	3	2.00
16	60–70	83	R	Trauma	0	0	5	0.67
17^a^	50–60	146	L	Trauma	3.3	1.7	2	2.33
18	60–70	517	R	Disturbed blood flow	0	0	3	1.00
19	50–60	72	L	Trauma and osteitis	3.5	3.5	1	2.67
20	50–60	484	L	Trauma	0	0	3	1.33
21	70–80	648	R	Trauma	0	0	3	3.00
22	60–70	32	R	Disturbed blood flow	0	0	4	0.33
23^*^	50–60	60	L	Trauma	0.9	0.9	1	1.33
24^*^	50–60	390	L	Trauma	4.8	4.8	1	3.33

L, left; R, right.

^*^Excluded from MEG analysis because MRI recording was not possible.

^a^No dipole solution could be determined; pain VAS_24_: assessment of PLP intensity during the last 24 h on a 10-cm visual analog scale with the two endpoints 0—“no” and 10—“unbearably strong”. Pain magnitude was calculated by dividing Pain VAS_24/_/Pain frequency; Pain frequency (1—“multiple times per day,” 2—“every day,” 3—“multiple times per week,” 4—“every week” or “multiple times per month,” and 5—“every month” or “less frequent”).

n.a., not applicable; MPI, pain intensity scale of the German Version of the West Haven-Yale Multidimensional Pain Inventory (MPI-D).

### 2.3. Assessment of phantom limb pain

PLP was operationalized by three variables. The average PLP intensity of the last 24 h (“How strong was your PLP during the last 24 h?”) was assessed using a 10 cm visual analog scale (VAS_24h_) with the two endpoints: 0—“no” and 10—“unbearably strong” (Scott and Huskisson, 1976; Price et al., 1983). Moreover, to support comparisons to the article by Makin et al. (2013b), we calculated the pain magnitude by dividing VAS_24h_ by frequency (1—“multiple times per day,” 2—“every day,” 3—“multiple times per week,” 4—“every week” or “multiple times per month,” and 5—“every month” or “less frequent”). To additionally measure pain intensity similar to Flor et al. (1995), we used the pain scale of the German version (MPI-D) of the West Haven-Yale Multidimensional Pain Inventory (Flor et al., 1990), which subsumes current pain, pain during the last week, and suffering from pain.

### 2.4. Stimulation procedure

The peroneal nerve was stimulated transcutaneously below the fibular head using a bipolar pad electrode (inter-electrode distance: 2 cm; Technomed Europe, Maastricht, Netherlands) to evoke somatosensory-evoked fields (SEFs). This stimulation is a standard technique for evaluating peripheral nerves and investigating the integrity of central somatosensory pathways (Cruccu et al., 2008). Standard techniques as used in this study assess the function of the dorsal column–lemniscal system, which projects from afferent nerve fibers via the dorsal column of the spinal cord to the cuneate nucleus and then as medial lemniscus to the thalamus and from there to the postcentral gyrus. Electrical stimulation preferentially activates large diameter myelinated afferent nerve fibers that project into the lemniscal system (groups I and II or Aα and Aβ) with conduction velocities of 30–80 m/s (Cruccu et al., 2008). Electrical stimulation was controlled by a Constant Current High Voltage Stimulator (DS7A; Digitimer, Hertfordshire, England) that delivered constant current wave pulses with a duration of 0.2 ms. The interstimulus interval varied between 700 and 1,400 ms. A ground electrode was placed around the thigh of the stimulated leg to reduce stimulus artifacts. Before each MEG recording, the individual stimulus intensity (motor threshold + ½ sensory threshold) was determined, producing a definite dorsal extension of the foot on the NAS. For controls, NAS and AS were assigned to the left or the right leg so that the proportion of right to left was similar to the patients. During MEG recordings, the subjects received a minimum of 350 stimuli per body side. The nerves were stimulated consecutively, starting with the NAS.

### 2.5. Data acquisition

Data were recorded with a 306-channel Neuromag Vectorview whole-head MEG system (Elekta Neuromag, Helsinki, Finland, Software Version 2.0). The 306 SQUIDs are divided into 102 magnetometers and 204 planar gradiometers, arranged as triple-sensor elements containing one magnetometer and two gradiometers each. The Neuromag system also includes a head position indicator (HPI) and a 3D digitizer. The sensors are arranged in a helmet-shaped dcSQUID sensor array (Elekta Neuromag User's Guide). The sampling frequency was 2,000 Hz, with the cutoff frequencies of the acquisition filters set to 0.1 and 660 Hz. To determine the exact head position, four coils were fixed to the subject's head (two behind the ears and two onto the forehead above the temples). A 3D Digitizer (3Space FASTRAK; Polhemus Inc., Colchester, USA) was used to digitize landmarks, coil positions, and the head shape of the scalp (with minimally 100 points), including points that mark the edge of the nose. Subjects were instructed to rest still and prevent head movements during MEG recording. The subjects wore non-magnetic clothes and removed all magnetic objects to exclude metal artifacts. The subjects were informed about the camera and microphone system, through which communication with the experimenter was possible at any time during the experiment.

ECG was recorded from the right collar bone and below the left costal arch. Before fixation, all electrode sites were cleaned using an electrolyte and peeling paste.

MEG recording was realized in the supine position of subjects, with their heads as close as possible to the MEG sensors. It was assured that the position of the subjects' heads was left-right symmetric and not tilted around the anterior–posterior axis. The backs of the subjects' heads rested on a cushion fixed to the helmet surface. MEG was recorded continuously. Epochs between −100 and 400 ms concerning stimulus onsets were used for further analysis. Head position was recorded continuously during the recordings (cHPI, Elekta Neuromag).

Additionally, a high-resolution (1 × 1 × 1 mm) T1-weighted MRI volume was obtained for each subject (3d-Flash-sequence, number of volumes: 192, TE = 3.03 ms, FOV = 192 mm, TR = 3,000 ms, slice thickness: 1 mm) in a 3T scanner (“Trio,” Siemens, Erlangen, Germany) to assist source analysis.

### 2.6. Data preprocessing

To reduce artifacts of brain signals, we used a time-domain extension algorithm (tsss, buffer length = 10 s, correlation limit = 0.9) implemented in MaxFilter (software version 2.2.10, Elekta Neuromag). MaxFilter utilizes the inherent RMS noise levels of the sensors. The gradiometer channel weight was set to 1, while the magnetometer channel weight was set to 100 (see version 2.2.10 of the recording software of Elekta Neuromag, 2005). MaxFilter included head movement correction with the window set to 200 ms, with a 10 ms step (ssst_mc, software version 2.2.10, Elekta Neuromag; Blume et al., 2014; Dietrich et al., 2017). Afterward, adjusted data were processed using Curry Neuroimaging Suite (version 7.0.9; Compumedics Neuroscan, Charlotte, USA). Data were band-pass filtered from 1 Hz (width 0.33 Hz) to 100 Hz (width 33 Hz), with a notch filter set to 50 Hz (width 5 Hz). Data were then automatically corrected for eye movement artifacts when the electrooculogram activity exceeded 150 μV and for heart activity using QRS-detection software implemented in Curry Neuroimaging Suite (Gratton et al., 1983). MEG signal amplitudes exceeding ± 3000 fT were excluded from further analysis. Artifact-free epochs were then averaged to calculate the SEFs. The mean numbers of averaged epochs for the patients (PG) were: *M*_PGas_ = 303.5, *SE*_PGas_ = 22.6; *M*_PGnas_ = 306.7, *SE*_PGnas_ = 23.6; and for controls (HC): *M*_HCas_ = 334.6, *SE*_HCas_ = 32.8; *M*_HCnas_ = 336.6, *SE*_HCnas_ = 35.9. An example of a somatosensory-evoked magnetic field in response to peroneal nerve stimulation and a field distribution is shown in Supplementary Figure 1.

### 2.7. Source reconstruction

The magnetometer and gradiometer were combined for analysis. The baseline was defined as −100 ms to −10 ms relative to stimulus onset. The coordinate system was defined by three landmarks (2× auricular points, 1× nasion), with the *x*-axis running from left to right, the *y*-axis from posterior to anterior (nasion), and the *z*-axis from inferior to superior (top). A realistic head model was defined for each subject using a boundary element model. A single equivalent current dipole (ECD) was fitted from 20 to 100 ms relative to stimulus onset. We chose the rotating dipole model (free dipole orientations; fixed dipole positions) within a time range of 4 ms around the first local maximum to include only stable solutions. Fitting criteria comprised maximal goodness of fit (GoF ≥ 70%), maximal dipole strength, and minimal confidence volume. We concentrated our analysis on the first component for the AS (Shimojo et al., 1996), for which we found a solution with a similar latency on the NAS.

### 2.8. Statistical analysis

All analyses were performed with IBM SPSS Statistics 23 (SPSS Inc., an IBM Company, Chicago, IL, USA).

We descriptively analyzed whether electrical transcutaneous stimulation of the peroneal nerve can elicit a somatosensory percept of the missing limb.

Furthermore, descriptive statistics were computed for ECD variables [coordinates (*x, y, z*), peak latencies, dipole strength, GoF, and confidence volume] to analyze whether fitting dipoles in the deprived cortex is possible.

Next, we explored whether the stimulation intensity for AS differs from NAS in amputees or controls. As stimulation intensities were not normally distributed, we used non-parametric tests instead of ANOVA. These included the Mann–Whitney test as a non-parametric test for comparison between groups and the Wilcoxon signed-rank test as a non-parametric test for comparison within groups. We tested whether values for patients' AS differ from NAS in patients and controls. To remove the influence of body fat on the stimulation intensities, we calculated the differences in stimulation intensity for the AS minus the stimulation intensity of the NAS as a variable of interest.

To explore whether the dipole characteristics of the AS of the patients differ from those of the NAS and controls, we first assessed the normality of the data by Kolmogorov–Smirnov tests. If variables were not normally distributed, we conducted a natural logarithm (ln) transformation of the respective variable and used the ln-transformed variable for further analysis. Separate two-factorial repeated-measures ANOVAs were performed for ECD variables [coordinates (*x, y, z*), peak latencies, dipole strength, and confidence volume] with the within-subject factor stimulation side (AS, NAS) and the between-subject factor group (patients, controls).

To explore whether PLP intensity was associated with (1) stimulation intensity and (2) dipole strength, we carried out Pearson's correlation analyses as two-tailed tests. Partial correlation was used to statistically remove the effects of stimulation intensity from the correlation between PLP intensity and dipole strength.

To select the sample size, we conducted *a priori* power calculations using the G^*^Power software (Faul et al., 2009). We assumed a medium-to-large effect concerning group differences. For repeated measurement ANOVAs given α = 0.05, power (1 – β) = 0.80, and effect size ρ = 0.5, total sample size was estimated to be *N* = 30, i.e., at least *N* = 15 in each group.

## 3. Results

### 3.1. Somatosensory percept of the missing limb

We first investigated whether eliciting a somatosensory percept of the missing limb is possible. The stimulation intensity of the AS produced a sensation that was rated as “comparable to the final stimulation intensity on the NAS” by all 24 patients. In 16 patients, the final stimulation intensity produced a visible twitch in the amputee's stump region distal to the position of the electrodes, and eight patients explicitly reported a movement in the phantom. No patient reported a painful sensation due to the applied stimulation intensity.

### 3.2. Equivalent current dipoles

**Figure 1:**
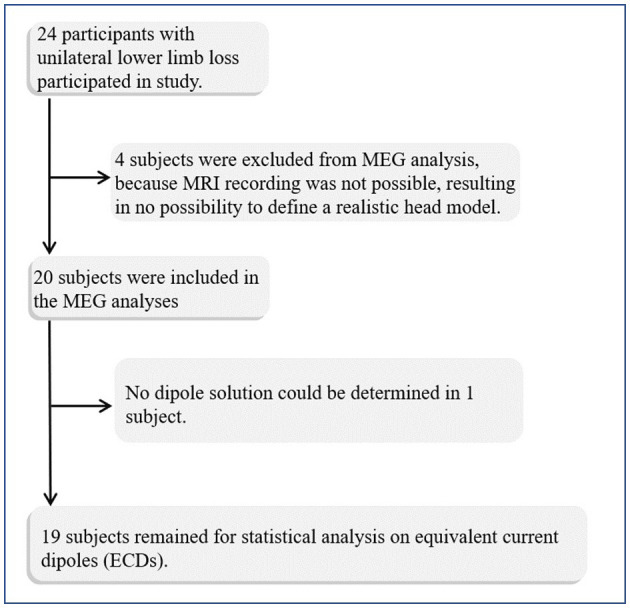
Flow chart of data acquisition and analysis.

**Figure 2:**
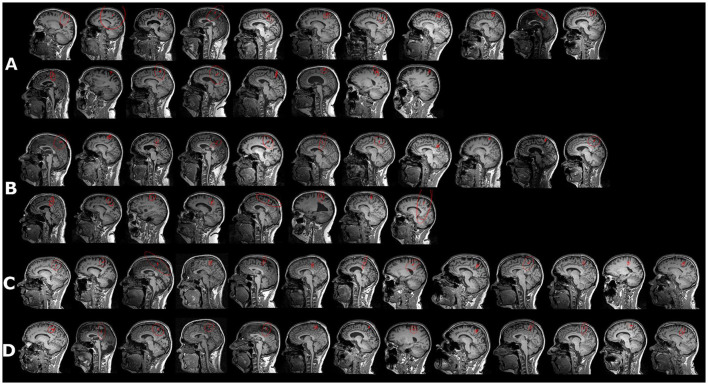
Equivalent current dipole (ECD) in response to peroneal nerve stimulation (red vector). **(A)** Affected side in patients, ordered according to pain magnitude, with the first picture in the first row displaying the person with the strongest PLP reported during the last 24 h; the second row displays subjects with no PLP during the last 24 h. **(B)** ECDs for the non-affected side in the same subjects as in **(A, C, D)**. Peroneal ECDs for healthy controls for both sides. For controls, NAS and AS were assigned to the left or the right leg so that the proportion was from right to left; however, the order of stimulation was similar to that of the patients. Red circle indicates confidence volume.

We also tested whether it is possible to fit dipoles in the deprived cortex. Four patients and one control were excluded from the source analysis due to missing MRI recordings and a realistic head model for the source analysis (Figure 1). ECDs for both stimulation sides were obtained for 19 out of 20 patients and for 14 controls (see Figure 2). No dipole solution could be determined in another patient, neither for the AS nor the NAS. Dipoles were localized on the mesial wall of SI. Descriptive statistics of ECDs are shown in Table 2. The median goodness of fit (GoF) of ECDs ranged from 95.1% in PG_AS_ to 97.2% in HC_NAS_, confirming a very good explanation of the variance of the measured magnetic fields.

**Table 2:** Descriptive statistics of ECDs and stimulation intensity.

	**Mean**	**SE**	**Range**
**Peak latency (ms)**			
Patients-AS	57.7	3.80	26.5–95.5
Patients-NAS	56.7	3.94	31.5–94.0
Controls-AS	52.6	4.04	33.5–81.5
Controls-NAS	49.2	3.48	34.5–76
**Abs(** ***x*** **)-coordinate (mm)** ^*^			
Patients-AS	10.9	1.80	0.1–27.8
Patients-NAS	11.5	1.54	0.3–26.7
Controls-AS	10.6	1.68	0.3–20.7
Controls-NAS	7.1	1.64	0.6–22.4
***y*** **-coordinate (mm)**			
Patients-AS	12.0	2.26	−3.5–30.7
Patients-NAS	12.0	2.20	−7.3–30.7
Controls-AS	9.74	2.13	0.2–29.3
Controls-NAS	8.4	2.25	−3.8–23.8
***z*** **-coordinate (mm)**			
Patients-AS	110.3	1.68	94.1–120.2
Patients-NAS	105.6	1.67	91.7–116.4
Controls-AS	104.1	2.01	88.0–115.5
Controls-NAS	105.6	2.41	91.8–118.9
	**Median**	**Interquartile range**	**Range**
**Goodness of fit (%)**			
Patients-AS	95.1	6.76	73.4–99.8
Patients-NAS	95.51	5.50	78.6–99.5
Controls-AS	97.2	4.05	92.4–99.2
Controls-NAS	97.2	4.14	91.1–99.0
**Confidence volume (ml/cm**3**)**			
Patients-AS	9.4	45.70	0.1–464.4
Patients-NAS	16.6	29.20	0.1–396.1
Controls-AS	8.9	37.35	0.6–22.4
Controls-NAS	16.2	22.65	0.2–96.6
**Dipole strength (**μ**Amm)**			
Patients-AS	30.7	65.00	5.2–324.0
Patients-NAS	62.00	95.1	9.4–322.0
Controls-AS	34.0	60.15	11.0–172.0
Controls-NAS	36.9	61.50	14.4–161.0
**Stimulation intensity (Hz)**			
Patients-AS	13.4	8.89	3.82–24.0
Patients-NAS	8.6	6.48	4.15–16.6
Controls-AS	7.2	2.19	1.9–13.6
Controls-NAS	6.1	3.31	3.5–12.6

PG, patients; HC, a group of healthy control subjects; AS, affected side; NAS, non-affected side; SE, standard error of the mean.

^*^x coordinates were analyzed using the absolute value (abs). For controls, AS and NAS were assigned to the left or the right leg, so that the proportion of right to left, therewith the order of stimulation, was similar to the patients.

### 3.3. Stimulation intensity

**Figure 3:**
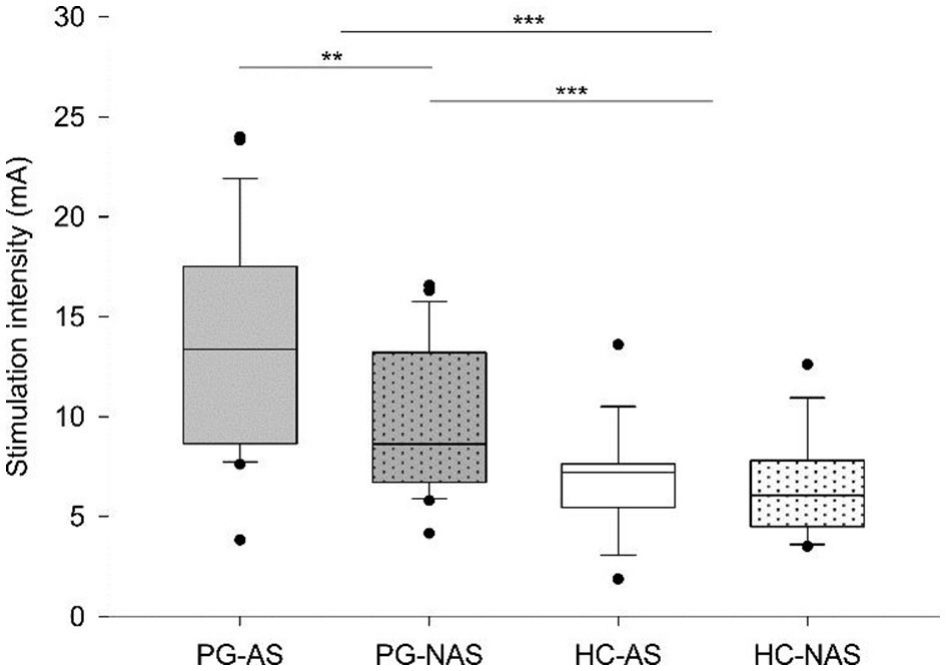
Stimulation intensities. Box plots show the median and interquartile range (Q25–Q75) of stimulation intensity for each stimulation side (affected side—AS, not affected side—NAS) in each group (PG—patients, HC—control group). Dots represent outliers outside the 5th−95th percentile. For controls, NAS and AS were assigned to the left or the right leg so that the proportion of right to left and, therefore, the stimulation order was similar to the patients. ***p ≤ 0.001, **p ≤ 0.01.

Then, we explored whether the intensity needed to stimulate similar somatosensory percepts in the truncated and intact peroneal nerve differs in amputees and controls. Table 2 shows descriptive statistics of the stimulation intensity. The Mann–Whitney test revealed significantly stronger stimulation intensities in the patients than in the controls (*Z* = −4.971, *p* < 0.001). To analyze this effect in detail, we compared the stimulation intensities of the AS and NAS in patients and the NAS between both groups (controls vs. patients). The Wilcoxon signed-rank test revealed significantly stronger stimulation intensities for PG_AS_ than for PG_NAS_ (*Z* = −2.886, *p* = 0.004). Accordingly, the Mann–Whitney test revealed stronger stimulation intensities in PG_NAS_ than in HC_NAS_ (*Z* = −3.412, *p* = 0.001; Figure 3).

### 3.4. ECD characteristics

We explored whether the dipole characteristics of the affected side (AS) of the patients differ from those of the intact side (NAS) and controls.

Differences in source localizations between AS and NAS and between patients and controls were evaluated by two-factorial ANOVAs for the following dependent variables: peak latency (ms), abs(*x*)-coordinate, *y*-coordinate, *z*-coordinate, confidence volume, and dipole strength. We used the absolute value (abs) for the *x*-coordinate (i.e., mirrored values for the left side), while the *y*- and *z*-coordinates were not transformed. We used log-transformed data for further analyses if the raw data were not normally distributed. All analyzed variables revealed no significant differences for Stimulation Side, Group, or Stimulation Side × Group when adjusted for multiple testing with *p* < 0.05/6 [peak latency: Stimulation Side (*F*_(1)_ = 4.631, *p* = 0.039), Group (*F*_(1)_ = 1.225, *p* = 0.277), Stimulation Side × Group (*F*_(1)_ = 1.133, *p* = 0.295); abs(*x*)-coordinate: Stimulation Side (*F*_(1)_ = 0.792, *p* = 0.380), Group (*F*_(1)_ = 1.739, *p* = 0.197), Stimulation Side × Group (*F*_(1)_ = 1.506, *p* = 0.229); *y*-coordinate: Stimulation Side (*F*_(1)_ = 0.127, *p* = 0.724), Group (*F*_(1)_ = 1.131, *p* = 0.296), Stimulation Side × Group (*F*_(1)_ = 0.166, *p* = 0.686); *z*-coordinate: Stimulation Side (*F*_(1)_ = 0.886, *p* = 0.354), Group (*F*_(1)_ = 2.031, *p* = 0.164), Stimulation Side × Group (*F*_(1)_ = 3.636, *p* = 0.066); confidence volume: Stimulation Side (*F*_(1)_ = 0.023, *p* = 0.881), Group (*F*_(1)_ = 0.108, *p* = 0.744), Stimulation Side × Group (*F*_(1)_ = 0.023, *p* = 0.880); dipole strength: Stimulation Side (*F*_(1)_ = 0.411, *p* = 0.526), Group (*F*_(1)_ = 0.688, *p* = 0.420), Stimulation Side × Group (*F*_(1)_ = 1.658, *p* = 0.207)]. Please note that the study was not powered for a MANOVA (or similar multivariate analysis). As a result, the study is unable to detect interdependencies among these outcome variables in the multiple ANOVAs.

### 3.5. Association of PLP intensity, stimulation intensity, and dipole strength

We explored whether dipole strength and stimulation intensity depend on PLP intensity.

#### 3.5.1. Association of PLP intensity and stimulation intensity

We separately correlated VAS_24h_, pain magnitude, and the pain scale of the MPI-D with the difference in stimulation intensity between AS and NAS in patients to investigate the association between stimulation intensity and PLP intensity as a marker of persisting pain and processing in the cortical representation. There was no significant association between stimulation intensity difference and PLP for all measures [VAS_24h_: *r*_(24)_ = −0.23, *p* = 0.29; pain magnitude: *r*_(24)_ = −0.12, *p* = 0.59; MPI (two missing values): *r*_(22)_ = −0.32, *p* = 0.15].

#### 3.5.2. Association of PLP intensity and dipole strength

There was no significant association between dipole strength and PLP when we controlled for differences in stimulation intensities [VAS_24h_: *r*_(16)_ = −0.045, *p* = 0.86; pain magnitude: *r*_(16)_ = −0.06, *p* = 0.82; MPI (two missing values): *r*_(14)_ = −0.15, *p* = 0.57].

Exploratory, the relationship between potential reorganization of the cortical representation and PLP intensity was analyzed. There was no association between the z-coordinate and PLP [VAS_24h_: *r*_(17)_ = −0.082, *p* = 0.738; pain magnitude: *r*_(17)_ = −0.184, *p* = 0.451; and MPI (two missing values): *r*_(15)_ = −0.173, *p* = 0.507] or between the Euclidian distances and PLP [VAS_24h_: *r*_(17)_ = 0.406, *p* = 0.085; pain magnitude: *r*_(17)_ = 0.317, *p* = 0.186; and MPI (two missing values): *r*_(15)_ = 0.416, *p* = 0.097].

## 4. Discussion

First, we found it possible to elicit a somatosensory percept of the missing limb by electrical transcutaneous stimulation of the peroneal nerve. Second, it is possible to fit dipoles in the deprived cortex. Third, exploratory analyses revealed that the intensity needed to stimulate similar somatosensory percepts in the truncated and intact peroneal nerve differs in amputees and in matched healthy volunteers. Fourth, the dipole characteristics of the affected side of the patients do not significantly differ from the intact side and controls. Fifth, dipole strength and stimulation intensity do not depend on PLP intensity in an exploratory analysis. The results are important for rehabilitation purposes and contribute knowledge about cortical representations of the affected lower limb in PLP patients.

In each patient, it was possible to stimulate the truncated peroneal nerve in a non-painful way so that the electrical stimulation induced a percept similar to the intact side. Such a procedure is usual in neurology. We used this kind of stimulation because we expected it to be feasible for many amputees and replicable. Another possibility to investigate the cortical representation of a lost limb percept is to stimulate referred sensation areas such as the stump or the cheek. Such a possibility was formerly shown, e.g., by Dietrich et al. (2018a). However, evoking referred sensations to investigate the lost limb representation can only be employed in amputees that present this phenomenon robustly. Knecht et al. (1996, 1998), who investigated the reliability of that phenomenon, reported considerable variance over time. The locations from which they could evoke referred sensations were constant for only a short period but differed considerably 4 weeks or 1.5 years later. Despite this, investigating the phenomenon of referred sensations could be a window into mechanisms that drive PLP (Weiss et al., 2022).

With peroneal nerve stimulation, we obtained ECDs in all but one patient. This result is in accordance with the findings of Mackert et al. (2003), who elicited cortical activities in response to the stimulation of peripheral deafferented arm nerves. However, our study differs from Mackert et al. (2003) findings in that we could localize the activity in the brain. In contrast, Mackert et al. (2003) assessed the primary component of the somatosensory-evoked potential (SEP) but did not localize the source in that detail. In this study, it is important to mention that the localization of sources was found at the expected contralateral site in SI, i.e., the mesial wall of the paracentral lobule, with no significant differences between affected sides and intact sides and no differences between patients and controls. The location of all dipoles is in line with a former study in controls (Shimojo et al., 1996), and it is in accordance with dipole locations in a previous study by our group (Dietrich et al., 2017). Therefore, the results of this investigation on leg amputees support the hypothesis that the cortical representations of missing lower limbs in SI are maintained.

Another difference from Mackert et al. (2003) is that we needed to adapt stimulation intensities to achieve a subjectively similar percept for both sides. Intensities were similar for both stimulation sides in Mackert et al. (2003). In contrast, we needed stronger stimulus intensities at the truncated nerve on the affected side compared to the intact side to receive similar percepts. Thereby, stimulation intensity on the intact side did not differ from the equivalent sides in controls. The short latency of the neural sources in response to stimulation and similarities in perceptions and SEF distributions suggest that our SEFs resulted primarily from stimulation directly at the truncated peroneal nerve. Presumably, the standard nerve stimulation technique used in this study did activate large diameter and myelinated primary afferent nerve fibers (groups I and II or Aα and Aβ) that project into the lemniscal system, as these afferents have the lowest threshold for electrical stimulation and larger conduction velocities (30–80 m/s); the stimulus did probably not activate small diameter and thinly myelinated or unmyelinated afferents (group III, or Aδ and group IV or C) because they have high thresholds for electrical stimulation and lower conduction velocities (2–33 m/s, 0.4–1.8 m/s) (Cruccu et al., 2008). The reasons why stronger stimulus intensities were required in patients to elicit a stimulus percept to the stimulation of the affected side that is similar to the intact side might be complex. Functional and structural degradation processes of peripheral nerve fibers or central representational areas are possible explanations. For peripheral nerves, several changes have been described in a seminal review by Flor et al. (2006), including alterations of neurons, axons, and loss of fibers. Soft tissue might replace lost fibers, resulting in a higher intensity to stimulate the necessary number of nerve fibers to receive the same percept. In addition to this, central representational areas have been found to undergo structural and functional degradation following amputation. Such alterations were observed in hand amputees who showed a reduced cortical volume of the hand area in SI/MI (Dettmers et al., 2001; Makin et al., 2013b), as well as reduced connectivity of the deprived area to other brain areas (Makin et al., 2013b; Preißler et al., 2013).

In addition to the contralateral responses in SI, an amputation-related reorganization was also found in the ipsilateral cortices. For example, Valyear et al. (2020) found that SI becomes responsive to cutaneous stimulation of the intact hand of amputees. This modality-specific reorganizational change persists for many years. They also found that the ipsilateral response is not associated with the tactile acuity of the intact hand, which leaves the functional relevance of ipsilateral responses unknown. In our study, we did not analyze ipsilateral responses. As MEG provides a high temporal resolution of SI activation, future studies could analyze the time course of activation in SI by using more complex models with multiple dipoles over time fitting in ipsilateral and contralateral cortices to provide further insights into this interesting question.

Our findings could also argue for the amputation of the hand because the basic structure of the nervous system is similar. Taking the results of Mackert et al. (2003) and our results together, one can conclude that the stimulation of truncated nerves leads to the activation of somatosensory SI representations of the lost limb. However, as SI representation is usage-dependent as well, the high dexterity of the upper limb must be associated with differences in sensorimotor representations compared to lower limb representations (see also Makin et al., 2013a).

While the findings of this study add information to our understanding of the potential to recover from deafferenting injuries, another window into the mechanisms of nervous system reorganization following amputation is investigating post-amputation SI changes after hand transplant (Frey et al., 2008; Philip et al., 2022). Frey et al. (2008) could show that the representation of a transplanted hand can recapture the pre-amputation SI hand territory when using palmar tactile stimulation delivered 4 months post-transplant. Interestingly, the evoked contralateral SI responses were indistinguishable in location and amplitude from those detected in healthy matched controls. Their results suggest that restoring afferent input to SI leads to the re-establishment of the gross hand representation within its original territory, even decades after amputation. Another case series by Philip et al. (2022) provided evidence that hand restoration patients show SI function within the range of both typical adults and amputees but with low-amplitude and individual-specific responses that indicate a wide range of potential cortical neurological changes following deafferentation and reafferentation. These results are in line with those of Blume et al. (2014, 2018), demonstrating changed cortical organization and considerable changes in somatosensory functions in patients after macroreplantation, i.e., patients who received their own upper limb after traumatic amputation. Further research is needed to clarify the mechanisms behind such heterogeneous processes.

The findings of our study suggest that nerve stimulation could be used for somatosensory feedback from a prosthesis to the deafferented leg nerves in the stump of amputees. It demonstrates that the truncated nerve may be used to establish both motor control and somatosensory feedback via the trunk when permanent functional connections between truncated nerves and interfaces to the prosthesis become available (Micera et al., 2010; Raspopovic et al., 2014).

Although the cortical representation seems preserved, its activation does not depend on PLP intensity. Makin et al. (2013b) reported that the pain magnitude was positively associated with activity in the deprived area of SI in a phantom movement task. In our study, neither the differences in stimulation intensities nor the dipole strength, i.e., the amount of cortical activation in response to peripheral stimulation, depended on the intensity of PLP. Possible reasons for this discrepancy might be differences in measurement and stimulation. Makin et al. (2013b) used fMRI, which integrates cortical activity over several hundreds of milliseconds, while we used MEG, i.e., the activity of one specific component of the SI activation with millisecond-precise resolution. Concerning stimulation, we used bottom-up stimulation coming from the peripheral nerve, while Makin et al. (2013b) used a top-down approach with a movement task. It is known that these different kinds of input use different activation patterns within the cortical layer architecture (Creutzfeld, 1983). These different activity patterns might lead to different gross activations within SI detectable by MEG.

There are limitations to this study. Transcutaneous nerve stimulation was used in this study. Direct nerve stimulation might elicit more naturalistic percepts (Graczyk et al., 2016) than transcutaneous peripheral nerve stimulation. However, this has yet to be shown for lower limb amputees. Moreover, the atrophy of the affected nerve might have influenced our results regarding the stimulation intensity. In future studies, it might be helpful for theoretical accounts of plasticity after amputation to measure cortical reorganization of neighboring areas at the same time as a persistent representation by combining the stimulation of the peroneal nerve with electrocutaneous stimulation of dermatomes that neighbor the deprived cortex (Dietrich et al., 2017). To account for the influence of body fat on stimulation intensity, we decided to use the difference between the affected side and the intact side (AS-NAS), following the assumption that this reduces interindividual variance due to body fat. Structural differences between affected and intact sides (e.g., atrophy at the stump) might influence the stimulation intensities needed and the results.

There are also other methodological limitations of this study. First, the sample is too small for confirmatory testing of differences and correlations. The decision to use individual ANOVAs was made after a thorough assessment of several factors, including the potential trade-offs associated with the complexity of the MANOVA approach. It is important to note that while MANOVA can offer insights into the overall multivariate pattern of results, it requires larger sample sizes due to the increased degrees of freedom associated with the joint analysis of multiple dependent variables. Considering our available resources and study objectives, we determined that conducting individual ANOVAs provided a more feasible approach. Moreover, as such an ANOVA approach is less conservative, the result concerning the still existing cortical representation of the amputated extremity holds true even with this less conservative testing. Second, while the spatial resolution of MEG analysis using a single equivalent dipole solution is quite good, simple, and accurate, it might need to explain the activation patterns more. A multi-dipole analysis might add such information. Third, our approach does not allow an analysis of other regions of interest in the brain that contribute to the processing of peripheral stimuli. Future studies with fMRI could be used to advance this knowledge.

Overall, the study shows that peripheral stimulation makes it possible to regain access to the deprived cortex after amputation. From a translational perspective, this provides hope that the truncated nerve may be used to establish both motor control and somatosensory feedback via the nerve trunk when a permanently functional connection between the nerve trunk and the prosthesis becomes available. Such functional connectivity might help to reduce PLP, as adding somatosensory feedback to prostheses has already been demonstrated to reduce PLP (Dietrich et al., 2012; Weiss et al., 2013). However, more research is needed to clarify the mechanisms of PLP (Ortiz-Catalan, 2018; Makin and Flor, 2020; Schone et al., 2022; Weiss et al., 2022). Alterations in the primary somatosensory cortex might play a smaller role than previously thought.

## Data availability statement

The raw data supporting the conclusions of this article will be made available by the authors, without undue reservation.

## Ethics statement

The studies involving humans were approved by Ethics Committee of the University Hospital Jena (No. 1312-05/04). The studies were conducted in accordance with the local legislation and institutional requirements. The participants provided their written informed consent to participate in this study.

## Author contributions

TW, WM, and GH conceived the investigation. CR, KB, SN, HK, and TW designed the experiment. CR, MG, KB, HK, and SN collected data. MG, HK, and CR analyzed data. CR wrote the first draft. All authors revised the manuscript critically for important intellectual content. All authors contributed to the article and approved the submitted version.
