# Supplementary material for: Stimulation of peroneal nerves reveals maintained somatosensory representation in transtibial amputees
Source: Front Hum Neurosci. 2023 Sep 7;17:1240937. doi: 10.3389/fnhum.2023.1240937 (PMC10512738; doi:10.3389/fnhum.2023.1240937)
Supplement: Supplementary file 1 [file Image_1.pdf]

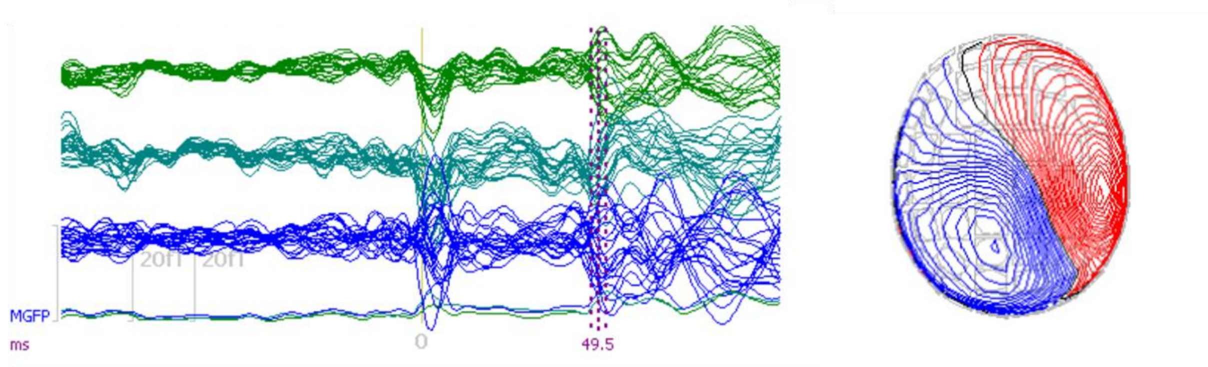

Supplementary Figure:

Left side: Magnetometer and gradiometer traces over time for a patient to stimulation of the peroneal nerve of the amputated extremity. The lower traces demonstrate the mean global field power (MGFP).

Right side: Distribution of the somatosensory evoked field at 49.5 ms. The distribution shows a bipolar distribution at this time point.
